# Supplementary material for: Loss of SETDB1-mediated H3K9me3 in human neural progenitor cells leads to transcriptional activation of L1 retrotransposons
Source: Nucleic Acids Res. 2026 Feb 5;54(4):gkag100. doi: 10.1093/nar/gkag100 (PMC12873604; doi:10.1093/nar/gkag100)
Supplement: gkag100_Supplemental_File [file gkag100_supplemental_file.pdf]

Supplementary Figures

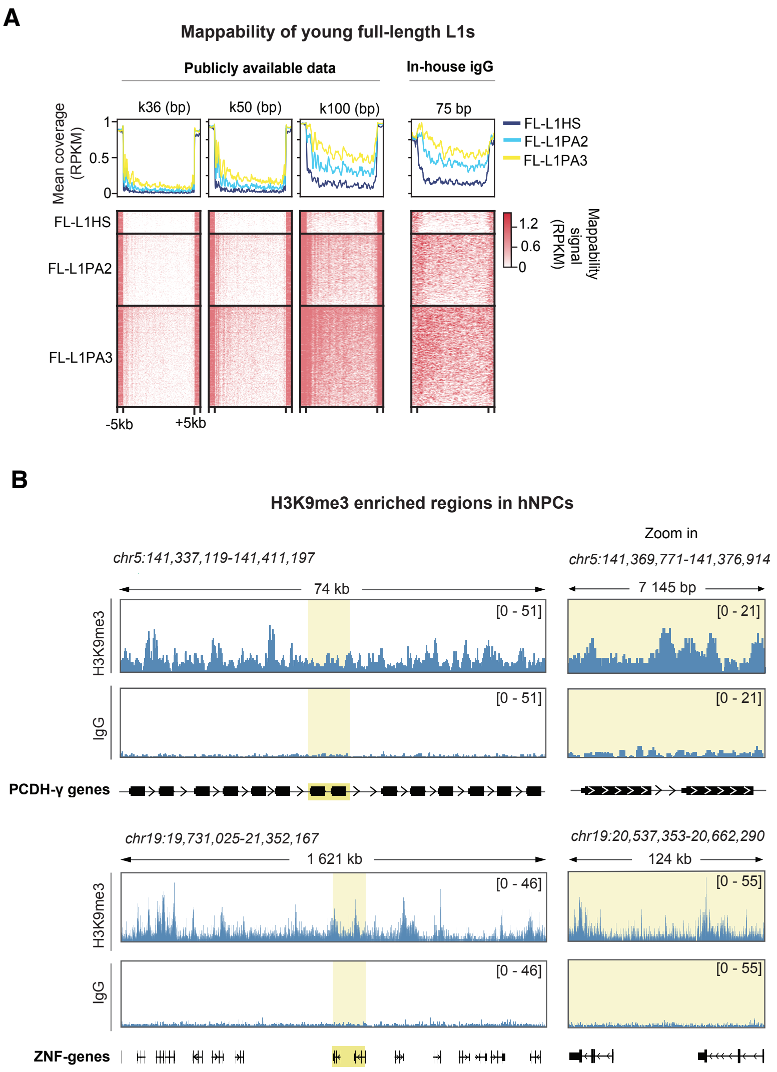

Supplementary Figure 1. H3K9me3 in hNPCs localizes to regions with well-established H3K9me3-enrichment A Mappability analysis of young full-length L1HS to L1PA3 using 36bp, 50bp and 100bp uniquely mappable kmers obtained from Karimzadeh et al, 2018, and an example of an in-house IgG experiment. **B** CUT&RUN analysis of H3K9me3 in hNPCs over genomic regions with well-established H3K9me3-enrichment; PCDH-ɣ cluster (top); KRAB-ZNF cluster (bottom).

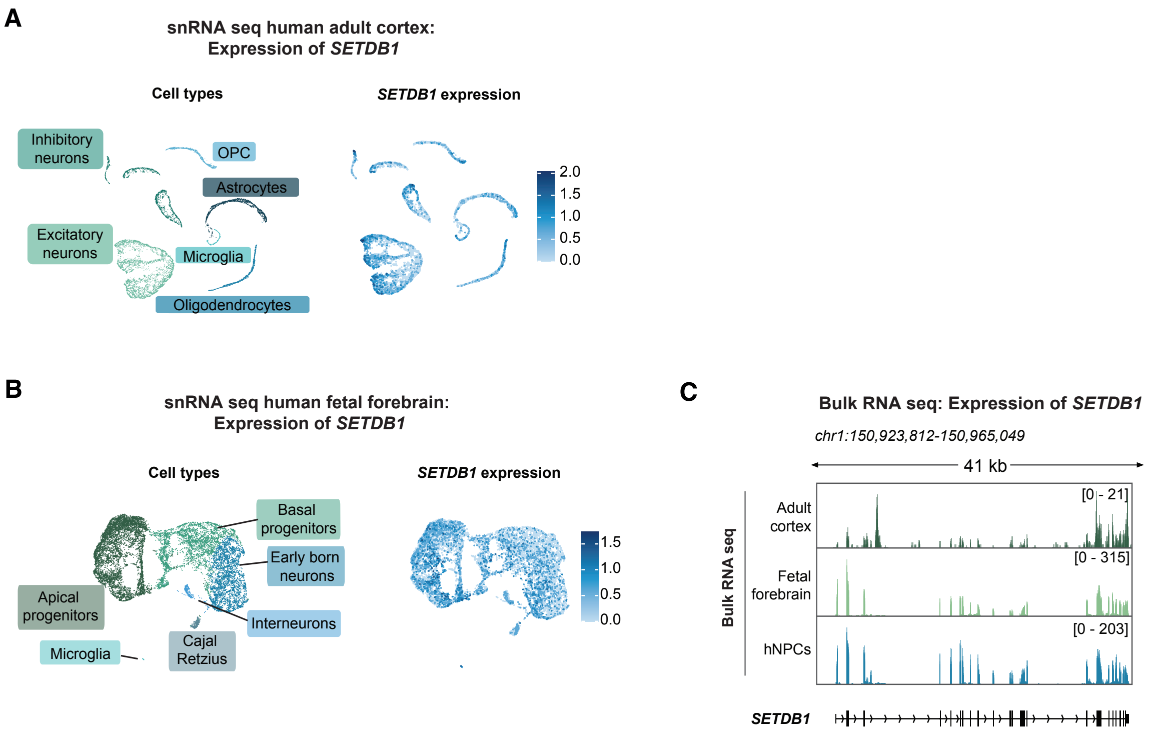

Supplementary Figure 2. SETDB1 is expressed in the adult and developing human brain and hNPCs A-B UMAP of snRNA-seq data showing SETDB1 expression in (A) human adult cortical tissue and (B) human fetal forebrain tissue. **C** Genome browser tracks showing bulk RNA sequencing data of SETDB1 expression in the human adult cortex, human fetal forebrain, and hNPCs. Data from Garza *et al.* (2023).

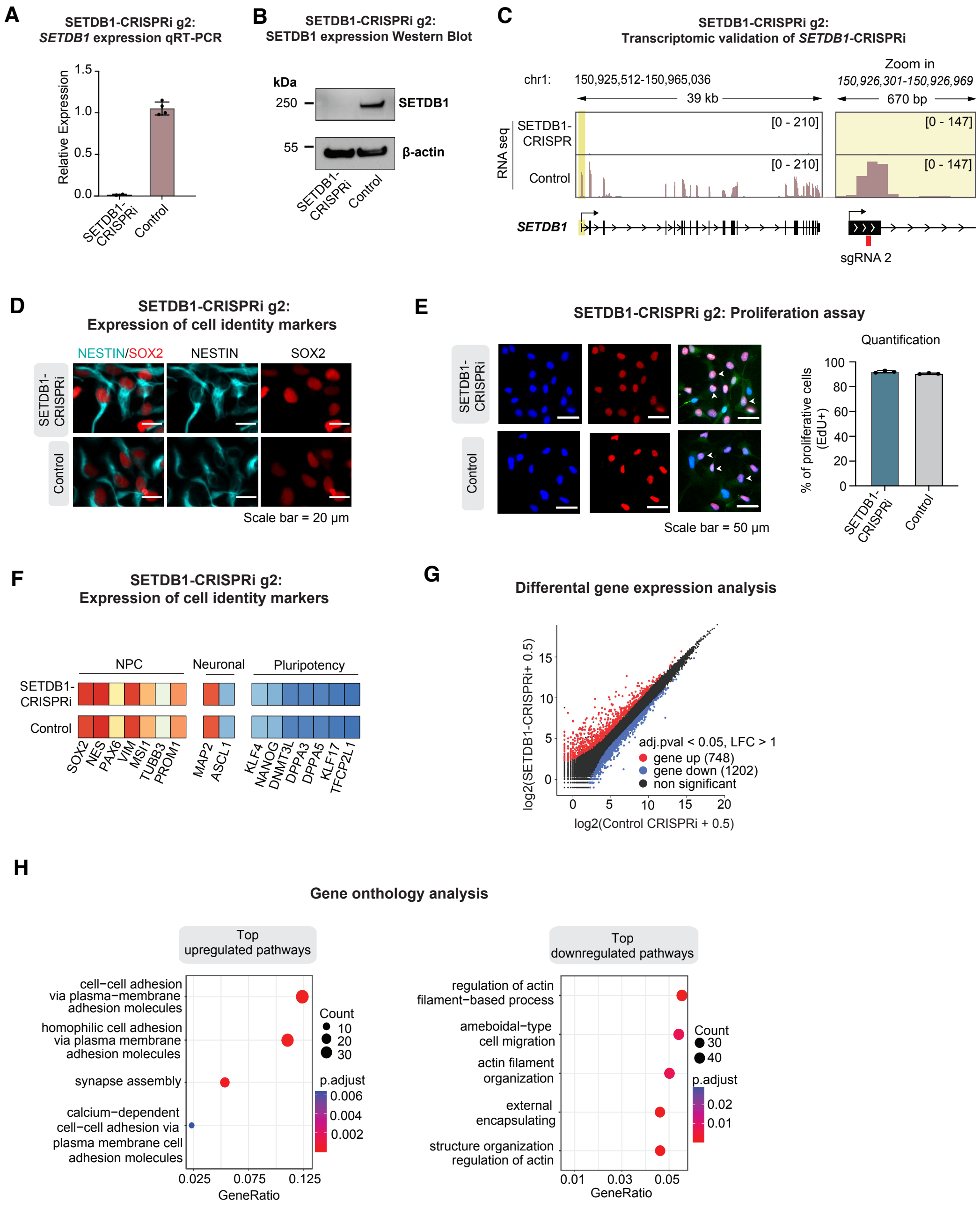

Supplementary Figure 3. Validation of SETDB1-CRISPRi using an alternative guide RNA A qRT-PCR analysis of SETDB1 expression after CRISPRi g2-based silencing in hNPCs. Results are shown as means with standard deviation (*n*=2 and *n*=4 respectively). **B** Western blot analysis of SETDB1 protein levels relative to β-actin protein levels in SETDB1-CRISPRi g2 and control hNPCs. **C** RPKM normalized genome browser tracks showing expression of *SETDB1* in SETDB1-CRISPRi g2 and control hNPCs as determined by bulk RNA sequencing **D** Immunocytochemistry of NESTIN (cyan) and SOX2 (red) in SETDB1-CRISPRi g2 and control hNPCs. Scalebar = 20 µm. **E** EdU proliferation assay showing cell proliferation in SETDB1-CRISPRi g2 and Control hNPCs. Scale bar=50 µm. **F** Heat map of NPC, neuronal and pluripotency gene marker mean expression in SETDB1-CRISPRi g2 (*n*=2) and control (*n*=4) hNPCs as determined by bulk RNA sequencing. **G** Mean plot showing differential gene expression in SETDB1-CRISPRi (*n*=4) compared to control (*n*=4) hNPCs. LFC >1, padj < 0.05 calculated with DESeq2. **H** Gene-ontology overrepresentation test of significantly differentially expressed genes (|LFC| > 1) associated with the selected top upregulated terms (left) and the selected top downregulated terms (right) in SETDB1-CRISPRi compared to control hNPCs.

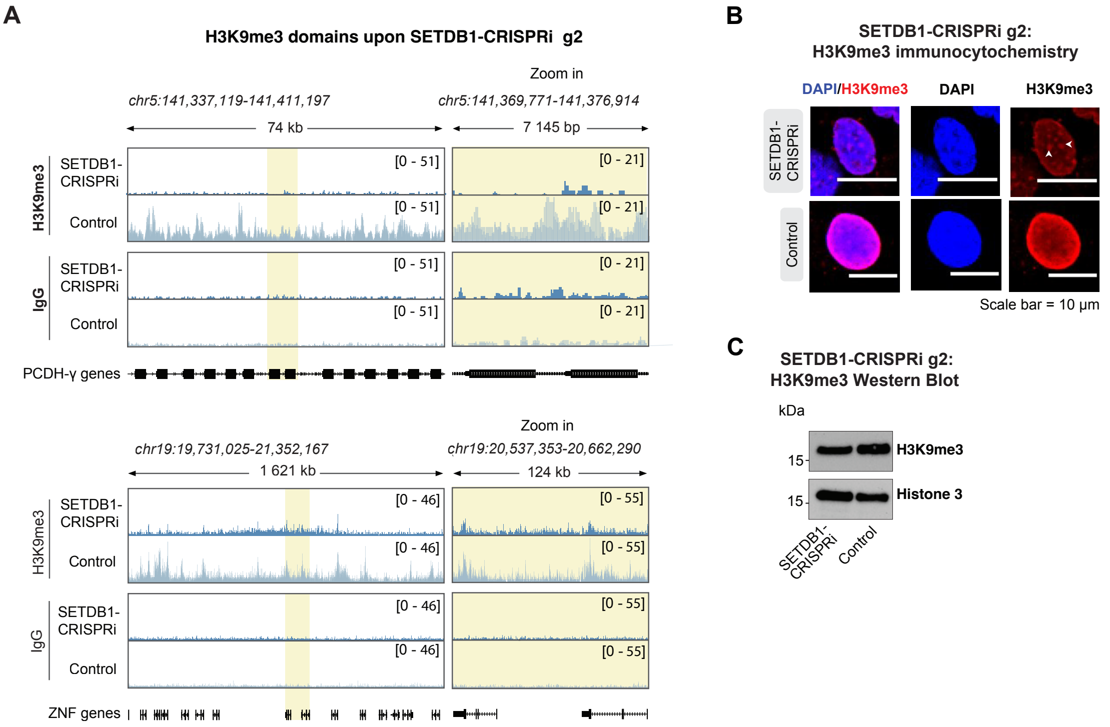

Supplementary Figure 4. H3K9me3 enrichment over genomic regions. **A** CUT&RUN analysis of H3K9me3 over genomic regions with well-established H3K9me3-enrichment in SEDTB1-CRISPRi and control hNPCs **B** Immunocytochemistry of H3K9me3 (red) and nuclear marker DAPI (blue) in SETDB1-CRISPRi g2 and control hNPCs. H3K9me3 foci are indicated with white arrows. Scalebar = 10 µm. **C** Western blot analysis of H3K9me3 levels relative to Histone 3 in SETDB1-CRISPRi g2 hNPCs compared to control.

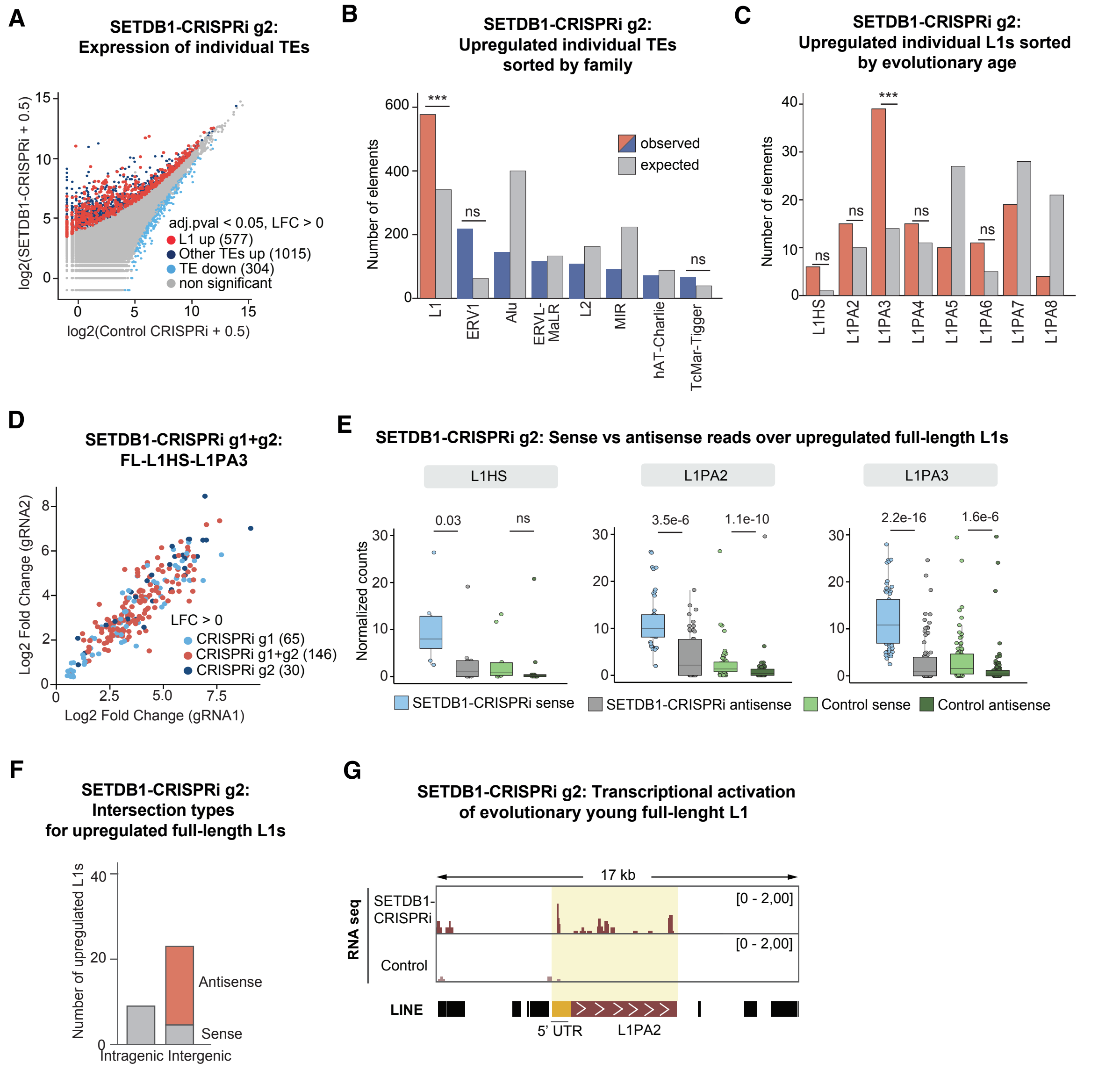

Supplementary Figure 5. Validation of transcriptional activation of L1s upon SETDB1-CRISPRi using an alternative guide RNA A Mean plot of uniquely mapped bulk RNA sequencing reads for SETDB1-CRISPRi g2 hNPCs (*n*=2) compared to control (*n*=2) over individual TEs categorized into L1 elements and other TEs. Log_2 fold change (LFC) >0, padj 0.05 calculated with DESeq2. **B** Observed vs expected number of individual transcriptionally activated elements per TE family in SETDB1-CRISPRi g2 hNPCs (n= 2) compared to control (n= 2). p-values were calculated using bootstrap-based empirical two-sided p-value calculation. **C** Observed vs expected number of transcriptionally activated L1 elements sorted by evolutionary age in SETDB1-CRISPRi g2 *(n*=2*)* compared to control hNPCs *(n*=2*)*. p-values were calculated using bootstrap-based empirical pvalue calculation. **D** Mean plot of uniquely mapped bulk RNA sequencing reads for SETDB1-CRISPRi g1 and g2 hNPCs (*n*=4) compared to control-CRISRPi over individual FL-L1HS-L1PA3 elements. Red dots indicate L1s significantly upregulated with both gRNAs, while blue and black dots indicate L1s significantly upregulated with either gRNA1 or gRNA2, respectively Log_2 fold change (LFC) >0, two-sided padj < 0.05 calculated with DESeq2. **E** Normalized read counts in sense and antisense for upregulated, full-length L1HS-L1PA3 elements in SETDB1-CRISPRi g2 (*n*=2) vs control hNPCs (*n*=4). Statistical significance calculated with Mann-Whitney U test. **F** Number, genomic location and direction of upregulated full-length (>6 kb) L1HS-L1PA3 elements in SETDB1-CRISPRi g2 (n=2) compared to control hNPC (n=2) ***G*** RPKM normalized genome browser tracks showing RNA-seq in SETDB1-CRISPRi g2 and control hNPCs.

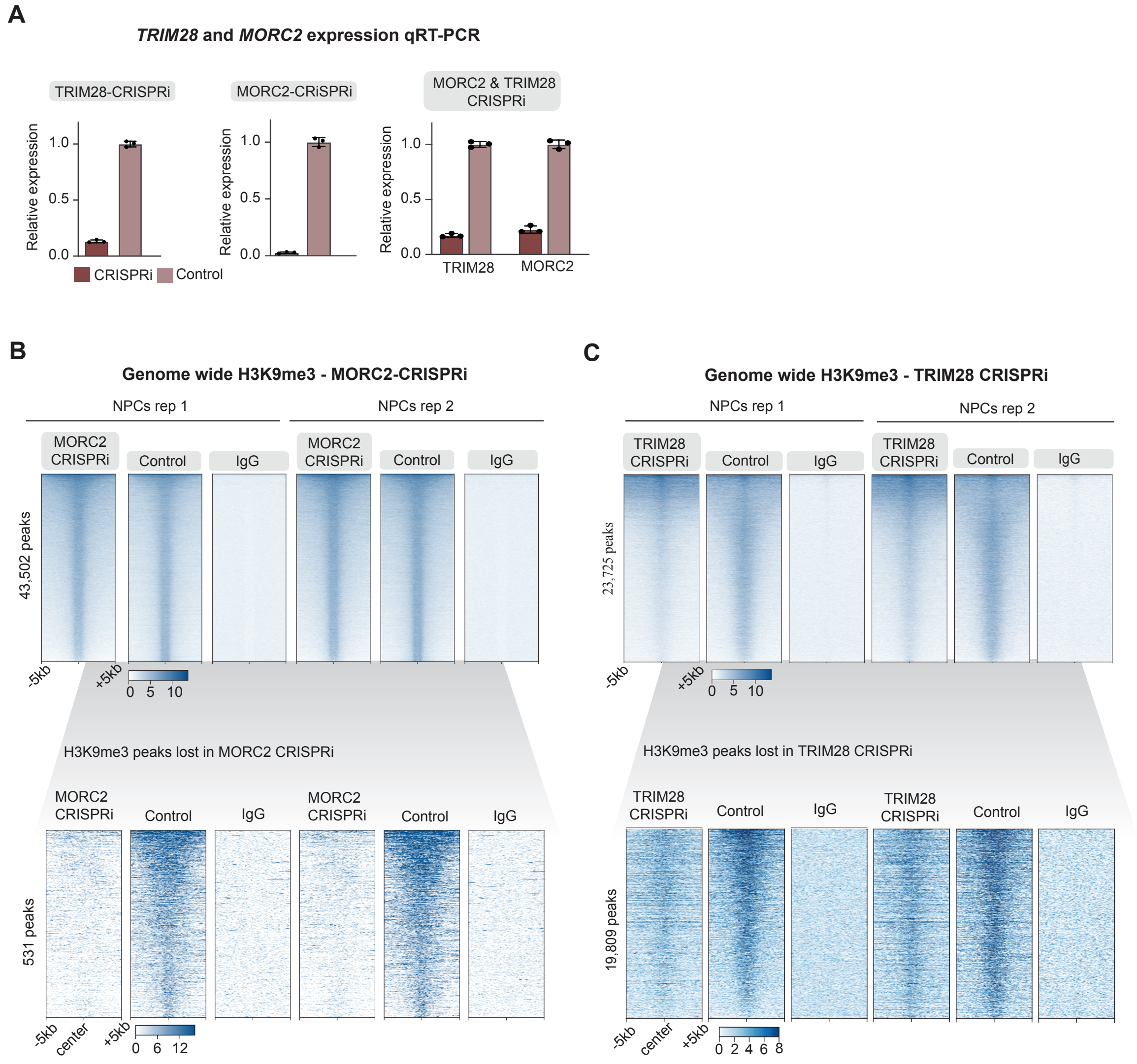

Supplementary Figure 6. SETDB1 regulates evolutionary young, full-length L1 elements independently from MORC2 and TRIM28. A qRT-PCR analysis of TRIM28 and MORC2 expression after CRISPRi based silencing in hNPCs. Results are shown as means with standard deviation (*n*=3). **B** RPKM normalized heat maps showing H3K9me3 CUT&RUN signal in MORC2-CRISPRi hNPCs genome-wide (top) and over genomic regions where H3K9me3 peaks were lost (bottom) compared to their respective controls and non-targeting control IgG. Data from Pandiloski et al. (2024)**. C** RPKM normalized heat maps showing H3K9me3 CUT&RUN signal in TRIM28-CRISPRi hNPCs genome-wide (top) and over genomic regions where H3K9me3 peaks were lost (bottom) compared to their respective controls and non-targeting control IgG. Data from Horvath et al. (2024).

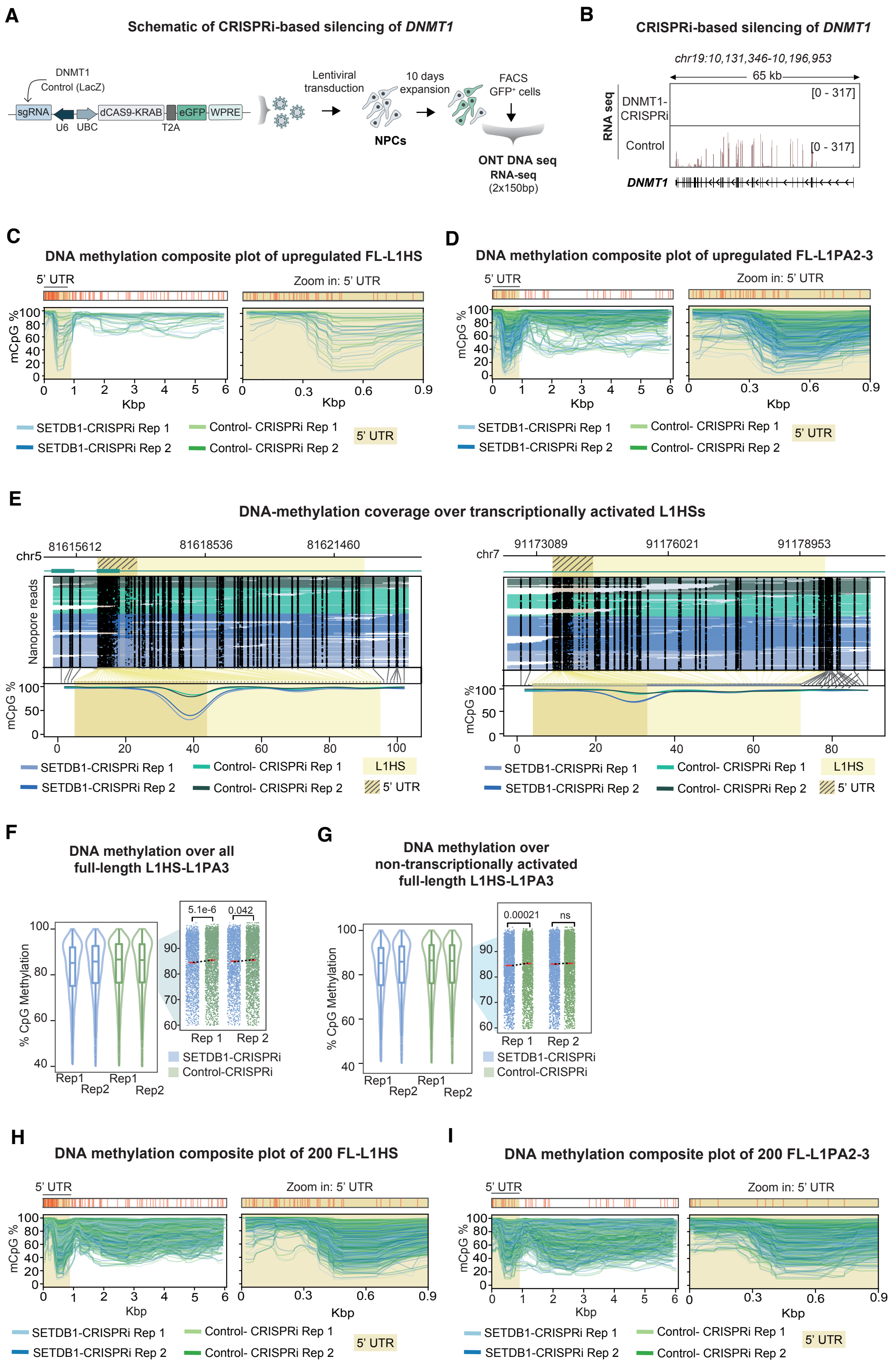
A
Schematic of CRISPRi-based silencing of DNMT1
DNMT1
Control (LacZ)
sgRNA
U6
UBC
dCAS9-KRAB
T2A
eGFP
WPRE
Lentiviral transduction
10 days expansion
NPCs
FACS GFP+ cells
ONT DNA seq
RNA-seq (2x150bp)
B
CRISPRi-based silencing of DNMT1
chr19:10,131,346-10,196,953
65 kb
RNA seq
DNMT1-CRISPRi
Control
[0 - 317]
DNMT1
C
DNA methylation composite plot of upregulated FL-L1HS
5' UTR
Zoom in: 5' UTR
mCpG %
Kbp
SETDB1-CRISPRi Rep 1
SETDB1-CRISPRi Rep 2
Control- CRISPRi Rep 1
Control- CRISPRi Rep 2
D
DNA methylation composite plot of upregulated FL-L1PA2-3
E
DNA-methylation coverage over transcriptionally activated L1HSs
chr5
81615612
81618536
81621460
chr7
91173089
91176021
91178953
Nanopore reads
L1HS
F
DNA methylation over all full-length L1HS-L1PA3
% CpG Methylation
5.1e-6
0.042
Rep1
Rep2
Rep 1
Rep 2
SETDB1-CRISPRi
Control-CRISPRi
G
DNA methylation over non-transcriptionally activated full-length L1HS-L1PA3
0.00021
ns
H
DNA methylation composite plot of 200 FL-L1HS
I
DNA methylation composite plot of 200 FL-L1PA2-3

Supplementary Figure 7. DNA methylation analysis over evolutionary young L1s A Workflow for CRISPRi-based silencing of *DNMT1* in hNPCs and downstream analysis. **B** RPKM normalized genome browser tracks showing *DNMT1* expression in DNMT1-CRISPRi and control hNPCs. **C** Composite DNA methylation profiles of upregulated full-length L1HS in SETDB1-CRISPRi (blue, n=2) and control (green, n=2) hNPCs. The 5' UTR is highlighted in yellow and a zoom in can be seen in the right panel. **D** Composite DNA methylation profiles of upregulated full-length L1PA2 and L1PA3 in SETDB1-CRISPRi (blue, n=2) and control (green, n=2) hNPCs. The 5' UTR is highlighted in yellow and a zoom in can be seen in the right panel. **E** Locus plot showing DNA methylation over upregulated L1HS in SETDB1-CRISPRi (blue, n=2) and control (green, n=2) hNPCs. Black dots indicate methylated CpGs, and methylation coverage of the L1 element can be seen at the bottom. The L1 element is highlighted in yellow, while the 5' UTR is marked with stripes. **F** Box plots of the methylation status over the promoter of all full-length L1HS, L1PA2 and L1PA3 elements in SETDB1-CRISPRi (n=2) and control (n=2) hNPCs. Zoom-in panels indicating mean methylation levels (red line) per condition for L1HS-L1PA3 elements. n values: L1HS = 306; L1PA2 = 972; L1PA3 = 1367. **G** Box plots of the methylation status over the promoter of full-length non-transcriptionally activated L1HS, L1PA2 and L1PA3 elements in SETDB1-CRISPRi (n=2) and control (n=2) hNPCs. Zoom-in panels indicating mean methylation levels (red line) per condition for L1HS-L1PA3 elements. n values: L1HS = 284; L1PA2 = 895; L1PA3 = 1225. **H** Composite DNA methylation profiles of 200 randomly picked full-length L1HS elements in SETDB1-CRISPRi (blue, n=2) and control (green, n=2) hNPCs. The 5' UTR is highlighted in yellow and a zoom in can be seen in the right panel. **I** Composite DNA methylation profiles of 200 randomly picked full-length L1PA2-L1PA3 elements in SETDB1-CRISPRi (blue, n=2) and control (green, n=2) hNPCs. The 5' UTR is highlighted in yellow and a zoom in can be seen in the right panel.

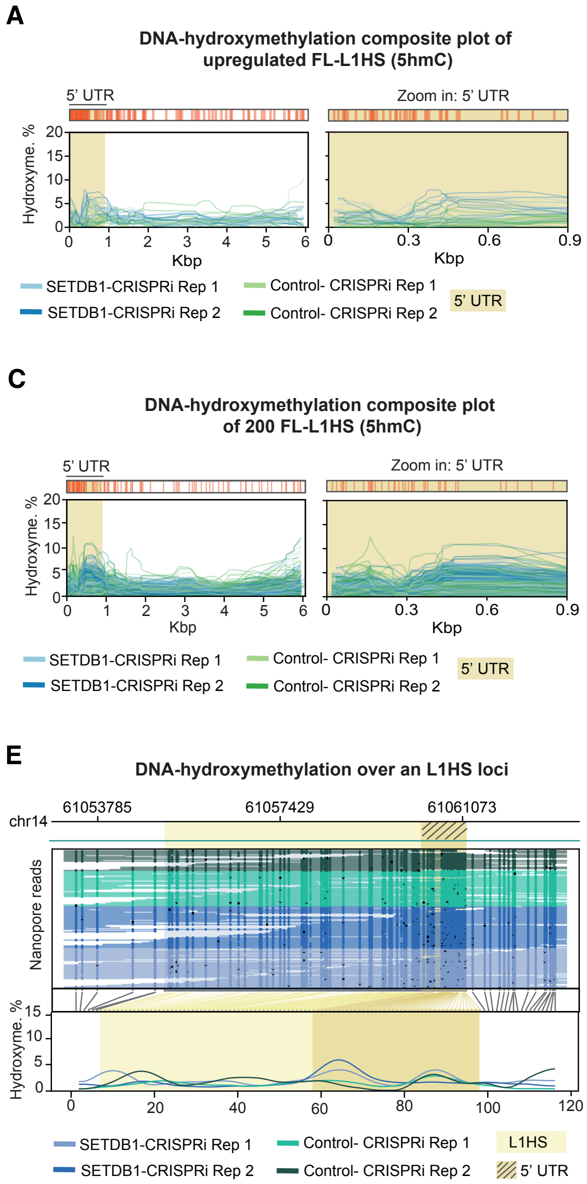

Supplementary Figure 8. DNA-hydroxymethylation over evolutionary young L1s A Composite DNA hydroxymethylation profiles of upregulated full-length L1HS in SETDB1-CRISPRi (blue, n=2) and control (green, n=2) hNPCs. The 5' UTR is highlighted in yellow. The 5'UTR is zoomed in in the right panel. **B** Composite DNA hydroxymethylation profiles of upregulated full-length L1PA2 and L1PA3 in SETDB1-CRISPRi (blue, n=2) and control (green, n=2) hNPCs. The 5' UTR is highlighted in yellow. The 5'UTR is zoomed in in the right panel..**C** Composite DNA hydroxymethylation profiles of 200 randomly picked full-length L1HS elements in SETDB1-CRISPRi (blue, n=2) and control (green, n=2) hNPCs. The 5' UTR is highlighted in yellow. The 5'UTR is zoomed in in the right panel. **D** Composite DNA hydroxymethylation profiles of 200 randomly picked full-length L1PA2-L1PA3 elements in SETDB1-CRISPRi (blue, n=2) and control (green, n=2) hNPCs. The 5' UTR is highlighted in yellow. The 5'UTR is zoomed in in the right panel.. **E** Locus plot showing DNA hydroxymethylation over upregulated L1HS in SETDB1-CRISPRi (blue, n=2) and control (green, n=2) hNPCs. Black dots indicate methylated CpGs, and methylation coverage of the L1 element can be seen at the bottom. The L1 element is highlighted in yellow, while the 5' UTR is marked with stripes.

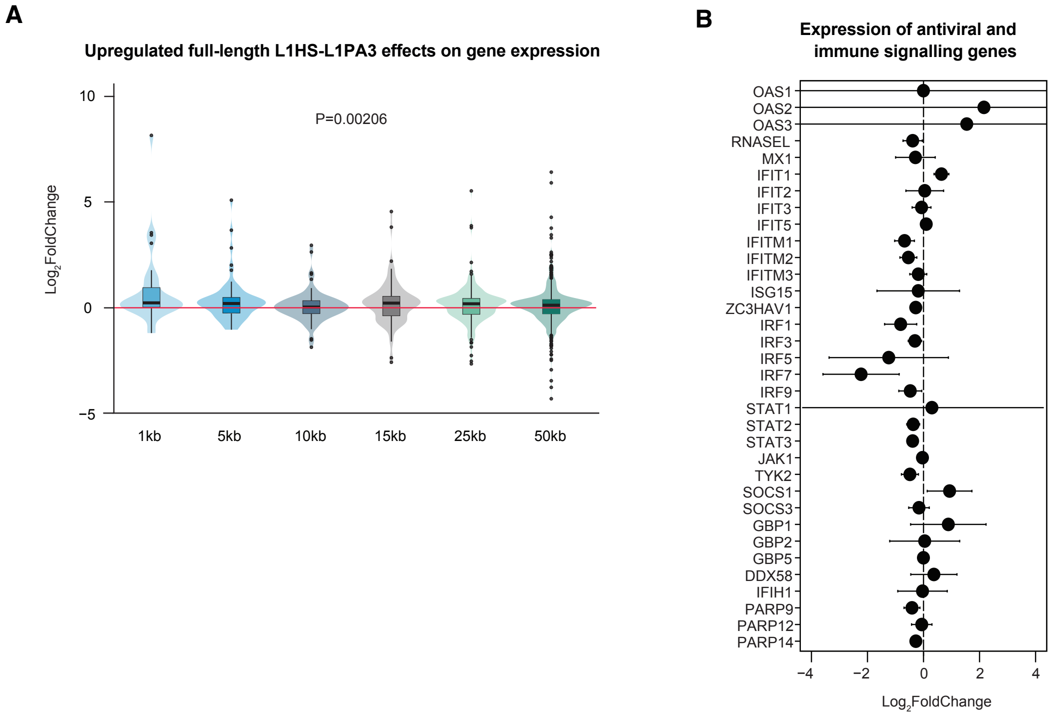

Supplementary Figure 9. The effect of upregulated evolutionary young, full-length L1s on nearby gene expression. A Violin plots showing the effect of upregulated full-length L1HS-L1PA3 elements on nearby gene expression (2-50kb) upon SETDB1-CRISPRi in hNPCs. p-value calculated with one-way ANOVA. **B** Differential expression analysis of selected antiviral and immune signaling genes in SETDB1-CRISPRi hNPCs (*n*=4) compared to control (*n*=4). LFC values are shown ± LFC standard error calculated by DESeq2.

Individual TE	Log2FoldChange	p-adjusted value
MER39B_dup439	12.5660730750503	1.8427529832473e-26
L2c_dup54301	12.2209237438216	4.09869177917656e-25
MER34B_dup283	11.7388139351731	2.93429232239475e-24
MLT1B_dup6701	11.1359864402149	4.05191990872606e-19
MIRb_dup150062	10.3913791660113	9.6211096384187e-19
MIRb_dup83332	10.3742843350223	4.39773387345283e-17
AluSx1_dup13234	10.3463773877304	2.18345277280823e-20
L2c_dup54302	10.0803146120823	2.89355744423237e-16
SVA_D_dup667	10.0527688112821	8.80024954039596e-19
MIR_dup66266	9.91282533692722	1.32675089321779e-16
Tigger1_dup11477	9.86311883738296	2.10590058343799e-18
LTR10C_dup335	9.64204668384122	1.93395417652645e-17
SVA_E_dup244	9.57541896859938	2.85991551525974e-12
LTR12C_dup2419	9.34014514735954	8.07950807168578e-14
THE1C_dup7048	9.17288181094644	1.27595527171481e-15
LTR10C_dup29	9.03749224093489	6.18977818132119e-15
L2c_dup96897	8.97118041487722	9.18595524144081e-15
THE1B_dup16084	8.73842550859459	5.00823323103967e-14
LTR79_dup800	8.51355207135647	7.37068570999639e-13
THE1C_dup7049	8.49481998710542	5.63699471444779e-13

Supplementary Table 1. Top 20 upregulated individual TEs upon SETDB1-CRISPRi Table showing top 20 differentially expressed individual TEs as determined by bulk RNA sequencing in SETDB1-CRISPRi (n=4) and control (n=4) hNPCs. LFC and padj calculated with DESeq2.
